# Coordinated alternation of DNA methylation and alternative splicing of PBRM1 affect bovine sperm structure and motility

**DOI:** 10.1080/15592294.2023.2183339

**Published:** 2023-03-03

**Authors:** Chunhong Yang, Yao Xiao, Xiuge Wang, Xiaochao Wei, Jinpeng Wang, Yaping Gao, Qiang Jiang, Zhihua Ju, Yaran Zhang, Wenhao Liu, Ning Huang, Yanqin Li, Yundong Gao, Lingling Wang, Jinming Huang

**Affiliations:** a Key Laboratory of Livestock and Poultry Multi-omics of MARA, Institute of Animal Science and Veterinary Medicine, Shandong Academy of Agricultural Sciences, Jinan, P. R. China; b Shandong Key Laboratory of Animal Disease Control and Breeding, Jinan, P.R.China; c College of Life Sciences, Shandong Normal University, Jinan, P. R. China

**Keywords:** Alternative splicing, dairy cattle, epigenetic regulation, *PBRM1*, spermatogenesis

## Abstract

DNA methylation and gene alternative splicing drive spermatogenesis. In screening DNA methylation markers and transcripts related to sperm motility, semen from three pairs of full-sibling Holstein bulls with high and low motility was subjected to reduced representation bisulphite sequencing. A total of 948 DMRs were found in 874 genes (gDMRs). Approximately 89% of gDMR-related genes harboured alternative splicing events, including *SMAD2, KIF17*, and *PBRM1*. One DMR in exon 29 of *PBRM1* with the highest 5mC ratio was found, and hypermethylation in this region was related to bull sperm motility. Furthermore, alternative splicing events at exon 29 of *PBRM1* were found in bull testis, including *PBRM1-complete, PBRM1-SV1* (exon 28 deletion), and *PBRM1-SV2* (exons 28–29 deletion). *PBRM1-SV2* exhibited significantly higher expression in adult bull testes than in newborn bull testes. In addition, *PBRM1* was localized to the redundant nuclear membrane of bull sperm, which might be related to sperm motility caused by sperm tail breakage. Therefore, the hypermethylation of exon 29 may be associated with the production of *PBRM1-SV2* in spermatogenesis. These findings indicated that DNA methylation alteration at specific loci could regulate gene splicing and expression and synergistically alter sperm structure and motility.

## Background

Semen quality is an important factor in the evaluation of bull fertility. The common parameters for evaluating semen quality include the semen volume per ejaculate, sperm concentration, sperm motility, post-thaw cryopreserved sperm motility, and sperm deformity rate [1]. Sperm motility is the main index used to assess the semen quality of a bull and determine whether semen can be frozen, affecting bull semen production and economic benefit. Sperm motility is also influenced by many factors, including genetic and epigenetic influences [2–5]. Therefore, the individual differences in semen quality should be identified precisely.

Although various methods are being used to assess bull semen quality from phenotypic and functional parameters, the accurate prediction of semen quality remains a major challenge. Recently, molecular mechanisms underlying the difference in semen quality have received considerable attention. One possible mechanism is DNA methylation, which could explain the phenotypic differences in sibling bulls. In the bull breeding programme, many full-sibling Holstein bulls were obtained via super-ovulation and embryo transfer. Despite being genetically similar, full-sibling bulls usually exhibit different semen quality phenotypes, including volume per ejaculate, sperm concentration, percentage of motility, progressive motility, mean path velocity, percentage of abnormal sperm, intracellular calcium and P25b levels [3,6,7]. Epigenetic regulation may play an irreplaceable role in semen quality. DNA methylation is a stable epigenetic modification of a genome and a transcriptional factor that regulates gene expression, which plays a role in cell reprogramming, tissue differentiation, and diseases [8,9]. The experimental design of twins plays an essential role in estimating the contribution to the inherent genomic sequence versus the epigenetic effects induced by environmental conditions in the expression of complex traits [10,11]. In the case of full siblings, we cannot completely rule out the influence of genetic components because they may have different genetic combinations. Nonetheless, the full-sibling Holstein bulls are an ideal model for investigating the relationship between DNA methylation and semen quality.

High-throughput genomic analyses, such as reduced representation bisulphite sequencing (RRBS), methylated DNA immunoprecipitation sequencing, or whole-genome bisulphite sequencing, allow the study of sperm DNA methylation from a genome-wide perspective. The relationship between DNA methylation patterns and sperm quality, including motility, morphology, and DNA fragmentation, was investigated in human [12–14] and cattle sperm [15]. The abnormality of sperm DNA methylation is closely related to bull semen quality (sperm morphology and motility) and fertility [16–18]. Several studies have revealed the hypomethylation pattern of the bull spermatozoa genome [19,20]. Furthermore, recent studies have shown that the sperm DNA methylation of the bull is closely related to sperm motility [21–23]. However, no functional evidence of the mechanism of DNA methylation in influencing spermatogenesis and sperm motility has been found.

Emerging evidence suggests that DNA methylation not only affects transcription, but also regulates alternative splicing (AS) [24–26]. The DNA methylation level of exons, particularly splicing sites, is higher than that of introns on both sides. About 22% of alternative exons are subjected to DNA methylation [24]. Although DNA methylation and AS regulation are common in spermatogenesis, to our knowledge, there is no evidence that the synergistic effects of DNA methylation and AS play a role in spermatogenesis.

We observed that three pairs of full-sibling Holstein bulls had similar genomic breeding values, but showed different sperm motility phenotypes. Therefore, we speculate that epigenetic mechanisms may play an important role. At the same time, considering the relationship between DNA methylation and AS, we further hypothesize that the synergistic regulation of DNA methylation and AS may be an important mechanism. The purpose of this study was to identify the genomic methylation sites that differ between full-sibling bulls with high and low sperm motility by RRBS, and to clarify the relationship between DNA methylation and AS in combination with previously obtained transcriptome data, and finally to find examples of methylation affecting gene splicing and expression, and thus influencing sperm structure and motility. Here, we provided evidence of the mechanism by which coordinated alterations of epigenetic regulation and RNA splicing of *PBRM1* promote spermatogenesis and semen quality.

## Materials and methods

### Animals and sample collection

Experiment 1: The same batch of fresh semen samples from three pairs of full-sibling Holstein bulls were collected to analyse the genome-wide sperm DNA methylation and identify differentially expressed mRNAs and lncRNAs using RNA-seq as described in our previous study [7]. Genomic breeding values (gEBV) were computed by the Animal Genetics Department of INRA (France National Institute for Agronomic Research; 14/3 release; https://idele.fr/detail-article/ibl-2014-8-evaluation-internationale-daout-2014). The gEBVs of the total merit index (called index de synthèse global in France or ISU) of each full-sibling Holstein bull pair obtained from Shandong OX Livestock Breeding Co., Ltd., were almost the same (Table S1), indicating that their genome information is similar, which can rule out the false detection of genomic DNA methylation caused by the genetic variation of C > T. The age of full-sibling Holstein bulls ranged from 4 to 6 years, with complete semen collection records, generally two times a week. Semen quality was evaluated in accordance with our national standards (NY-1234-2018) [27]. Among them, sperm motility assessment method is as follows. The semen and diluent were raised 5–10 μL on the slide, covered the slide, and the motility was examined by using an Olympus phase-contrast microscope at 37°C constant temperature to obtain the subjective assessment data. Based on the average sperm motility calculated by counting the semen quality records of the last two years, three pairs of full-sibling Holstein bulls, two of which had different sperm motility, were obtained and allocated into the high motility group (hereafter referred to as H1, H2, and H3; average sperm motility = 68.7%; average ISU = 141) and the low motility group (referred to as L1, L2, and L3; average sperm motility = 60%; average ISU = 140.7; Table S1). Bulls with sperm motility above 68% were defined as the H group, whereas those with sperm motility below 62% were defined as the L group, and the difference in sperm motility between the paired individuals was at least 8 percentage points. Three millilitres of fresh semen sample of each bull was collected. After evaluating semen quality, each semen was washed with PBS three times, and the pellet was resuspended in 1 mL of PBS and mixed gently. Each sperm suspension was divided into two parts, one for DNA extraction for RRBS and bisulphite sequencing PCR (BSP) analyses and the other for RNA extraction for RNA-seq [7].

Experiment 2: Fresh semen samples collected from about 3-year-old adult bulls (n = 6) were used to prepare the semen smear. The fresh semen was diluted, and 10 μL of fresh semen was placed and evenly spread onto a slide, dried at room temperature for 2–3 h, and used for immunofluorescence (IF) staining or stored at −80°C for future experiments.

Experiment 3: Testes were collected from 12 Holstein bulls at varying ages, including 2 days old (abbreviation 2 D, n = 3), 12 months old (12 M, n = 3), 2 years old (2 Y, n = 3), and 4 years old (4Y, n = 3), and each age stage included three bulls. The collected testes were quickly stored in liquid nitrogen to extract total RNA and protein for qRT-PCR and Western blot, respectively.

All protocols for collecting samples from bulls were reviewed and approved by the Animal Care and Use Committee of Shandong Academy of Agricultural Sciences. The experiment was conducted under the regulations and guidelines established by this committee.

### RRBS

Bull sperm DNA was extracted using a high-concentration salt protocol [28]. The quantity and quality of purified DNA were assessed using Nanodrop ND-2000 (Thermo Fisher Scientific, Waltham, USA). Genomic DNA methylation was sequenced using RRBS [29,30]. In brief, an appropriate amount of sperm genomic DNA with A260/280 ratio of 1.8:2.0 was digested using the restriction enzyme MspI (New England Biolabs, MA, USA), which specifically recognized shear C|CGG sequences. In addition, this enzyme was not sensitive to methylation modification. The T4 DNA polymerase and Klenow enzyme were used to flatten the hanging structures with different sizes into flat ends. The 300–400 bp DNA fragment was screened and purified using Agencourt AMPure XP beads (Beckman, USA). A base was added to the flat end of the sequence. The DNA fragments with a sticky end were attached to the methylated junction sequence using the Paired-End DNA Sample Prep kit (Illumina) and purified. Therefore, 40–220 bp enzymatic fragments were cut for recovery by using gel electrophoresis and treated with bisulphite to change the unmethylated C into T, whereas the methylated C remained unchanged. These fragments were amplified and purified using the MinElute PCR Purification kit (Qiagen, Germany). Finally, paired-end 2 × 150 bp sequencing was performed using the Illumina Hiseq 4000 platform (Hangzhou Lianchuan Biotechnology Co., Ltd.).

### RRBS data analysis

The raw data of RRBS has been uploaded to NCBI SRA, and the accession number was PRJNA818321. Raw sequencing data were filtered and assembled, and low-quality reads (Q ≤ 10 and N > 5%) were filtered out from raw reads. Original and effective sequencing quantities were counted, and Q20, Q30, and GC contents were comprehensively evaluated. High-quality and clean reads were mapped onto the cattle reference genome (Bos_Taurus UMD3.1) using the Bowtie2.1.0 program in the BSgenome software package to ensure accurate and reliable analysis results [31].

The methylation level of 5mC (CpG, CHG, and CHH; H = A/C/T) was classified on the basis of its location, including the promoter, exon, intron, and intergenetic regions. DMRs were determined using the R package-Methyl Kit with default parameters (1000 bp slide windows, 500 bp overlap, *P* value ≤ 0.05) [32]. The average ratio of 5mC in every DMR of each group was calculated. DMR with |log2FC| ≥ 1 and *P* ≤ 0.05 was considered significant. DMR-related genes (DMGs) were clustered on the basis of the Gene Ontology (GO) annotation database and KEGG pathway analysis through DAVID (https://david.ncifcrf.gov/) and Pathview (https://pathview.uncc.edu/) [33,34].

### BSP

In addition, 500 ng of genomic DNA from each fresh semen sample was treated using the BisulFlash DNA modification kit (Epigentek, USA) under the manufacturer’s protocol [35]. The BSP primers of DMGs were designed using Methyl Primer Express Software v1.0 (https://www.urogene.org/methprimer/, Table S2). PCR was performed using the *TaKaRa LA Taq®* (Code No. RR02MA, TAKARA, Japan), and the following components were mixed to prepare the PCR reaction: 0.25 μL of *TaKaRa LA Taq* (5 U/μL), 2.5 μL of 10× LA Taq Buffer II (Mg^2+^ Plus), 4 μL of dNTP Mixture (2.5 mM each), 1 μL of BS conversed genomic DNA, 1 μL of PBRM1-BSP-F/R, and 15.25 μL of ddH_2_O. The PCR reaction was set as follows: 94°C 1 min, 35 cycles of (98°C, 10s; 68°C, 15s and 72°C, 15s), 72°C 10 min, 4°C hold. PCR products were purified using the Gel/PCR extraction kit (Tiangen, Beijing, China), cloned into the pEASY-T3 vector (TransGen, Beijing, China), and transformed into *Escherichia coli* DH5α cells for clone sequencing. Only the sequences derived from clones with > 95% cytosine conversion were analysed. The percentage of DNA methylation was calculated by counting the number of methylated CpGs from the total number of CpG sites in individual clones. The reference sequence of a specific gene was used as a reference for methylation status analysis by using BiQ Analyser v0.7.

### qRT-PCR

Total RNA was extracted using the TaKaRa MiniBEST Universal RNA Extraction Kit (TaKaRa, Dalian, China). RNA was quantified and quality assessed using Nanodrop ND-2000. Complementary DNA was synthesized using the PrimeScriptTM RT reagent Kit with gDNA Eraser (TaKaRa, Dalian, China). The qRT-PCR primers (Table S2) were designed using Primer-blast (NCBI) and performed using TB Green Premix Ex Taq (RR820A, TaKaRa, Dalian, China). The reaction system contains 10 μL of TB Green Premix Ex Taq II (Tli RNaseH Plus, 2×), 0.8 μL of PCR Forward Primer (10 μM), 0.8 μL of PCR Reverse Primer (10 μM), 2 μL of cDNA (<100 ng), and 6.4 μL of DEPC water, reaching 20 μL. qPCR was performed on LightCycler 480 II, and the PCR amplification procedure was as follows: 95°C for 30s to activate the reaction and 95°C for 5 s and 60°C for 30s for 40 cycles. The collection of the melting curve was performed in accordance with the program of the instrument. Raw qRT-PCR Ct values were normalized against the geometric mean of the *β-actin* gene [6,7]. The 2^−ΔΔCt^ method was applied for calculating the relative gene expression level [36]. Each experiment was performed in triplicate.

### Identification of AS events associated with sperm motility

The RNA-seq data of six semen samples of Holstein bulls were obtained from our previous study (NCBI SRA database, accession number SRP158901), whose semen samples were of the same batch as those used in RRBS [7]. Qualitative analysis and statistics of AS events of the transcriptome of each sample were performed using ASprofile (https://ccb.jhu.edu/software/ASprofile/) based on the gene model predicted by Cufflinks. AS events were primarily divided into five classical types: including or excluding single (SKIP_ON/OFF) and multiple exons (MSKIP_ON/OFF), including or excluding single (IR_ON/OFF) and multiple (MIR_ON/OFF) introns, alternative exon (AE) ends, alternative TSS, and alternative transcription termination site (TTS, Fig. S1). The other five types belong to fuzzy boundary shear types, including approximate SKIP (XSKIP), approximate MSKIP (XMKIP), approximate IR (XIR), approximate MIR (XMIR), and approximate AE (XAE). The difference in AS transcript expression between the H and L groups with |log2FC| ≥ 1 and *P* < 0.05 was considered significant.

### Western blot

The total protein of the bull testis was extracted using RIPA lysis buffer (including 1% PIC) and separated using 10% SDS–PAGE. Immunoblot analyses were performed using rabbit anti-PBRM1 (1:1000, ab196022, Abcam), β-actin rabbit (1:10,000, AC026, ABclonal), or anti-α-tubulin mouse mAb (1:5000, AC012, ABclonal) with horseradish peroxidase-conjugated secondary antibodies (goat anti-rabbit IgG, ZB-2301 ZSGB-BIO; goat anti-mouse IgG, ZB-2305, ZSGB-BIO). Immobilon® Western Chemiluminescent HRP Substrate was used as the chemiluminescence detection reagent (P90719, Millipore, USA).

### Immunofluorescence

For IF staining of PBRM1, the fresh bull semen spread onto polylysine-coated slides was air dried, fixed using 4% paraformaldehyde for 5 min, washed three times in PBS, permeated in 0.2% Triton X-100 for 15 min, and blocked using 5% BSA for 1 h at room temperature. After washing with PBS three times, the samples were incubated with primary antibodies diluted with PBS (including anti-rabbit PBRM1 [1:100] (AB196022, Abcam, Cambridge, UK) or mAb 414 antibody (1:100, 902,097, BioLegend, San Diego, USA)) at 4°C overnight. Then, samples were incubated with secondary antibodies, including Alexa Flour® 488-conjugated goat anti-rabbit IgG (1:150; ZF-0511, ZSGB-BIO, Beijing, China) for 1.5 h at 37°C. For co-localization analysis of the nuclear pore complex (NPC) and PBRM1, the mAb 414 antibody was visualized by using a TRITC-conjugated secondary antibody, and PBRM1 labelling was visualized by using a FITC-conjugated secondary antibody. The samples were incubated in DAPI staining solution (C1005, Beyotime, China) for 10 min at room temperature and washed three times with PBS. The samples were added with an anti-fluorescence quencher and placed in a cover glass, and the edge was sealed with nail oil. Immunofluorescence staining was imaged at 200× magnification using an inverted fluorescent microscope (IX73, OLYMPUS, Japan).

### Transmission electron microscopy and immunoelectron microscopy

The ultrastructure of sperm was detected by TEM. Based on the density of fresh bull semen, approximately 5 × 10^6^ sperm was transferred into a 1.5 mL tube and then centrifuged at 4000 × g for 2 min to obtain a pellet. The pellet was fixed in 2.5% glutaraldehyde with 0.1 M cacodylate buffer (pH 7.4), post-fixed with 1% OsO_4_, dehydrated through a graded series of ethanol solutions to 100% ethanol, and embedded in London Resin (LR) White. Thin sections (50–70 nm) were cut on Leica EM UC7 ultramicrotome (Leica, Germany) and stained with uranyl acetate and lead citrate. All specimens were examined using a transmission electron microscope (HT7800, HITACHI, Japan) at 80 kV.

For electron microscopic immunolocalization, the bull sperm pellet was fixed with 4% paraformaldehyde and 0.01% glutaraldehyde for 12–14 h, washed with PBS five times at 4°C (15 min/time), and then dehydrated through a graded series of ethanol solutions to 100% ethanol. The sample was infiltrated with and embedded in LR white, which was polymerized by UV light for 24 h at −20°C. Thin sections (50–70 nm) were cut using a Leica EM UC7 ultramicrotome (Leica, Germany) and scooped up with nickel mesh. Then, slices were blocked with 1% BSA in PBS and probed with an anti-PBRM1 monoclonal antibody overnight at 4°C. After washing with PBS, sections were incubated for 1 h at room temperature using a 10 nm gold-conjugated secondary antibody, namely, goat anti-rabbit IgG. The sections were washed with distilled water and stained with uranyl acetate and lead citrate before the examination.

### Statistical analysis

Statistical significance of semen quality was measured using Student’s paired t-test. The statistical significance of the gene expression of qRT-PCR and the methylation level of BSP were tested using the Linear Model in the SAS v9.0, and comparisons were performed using Duncan’s multiple-range test. In addition, the *P* value was adjusted using the Bonferroni method. Chi-square test was used to check the association between loss expression of PBRM1 and sperm tail breakage. A *P*-value less than 0.05 was considered statistically significant.

## Results

### Genome-wide DNA methylation was different between high- and low-sperm-motility groups of bull

**Figure 1.**
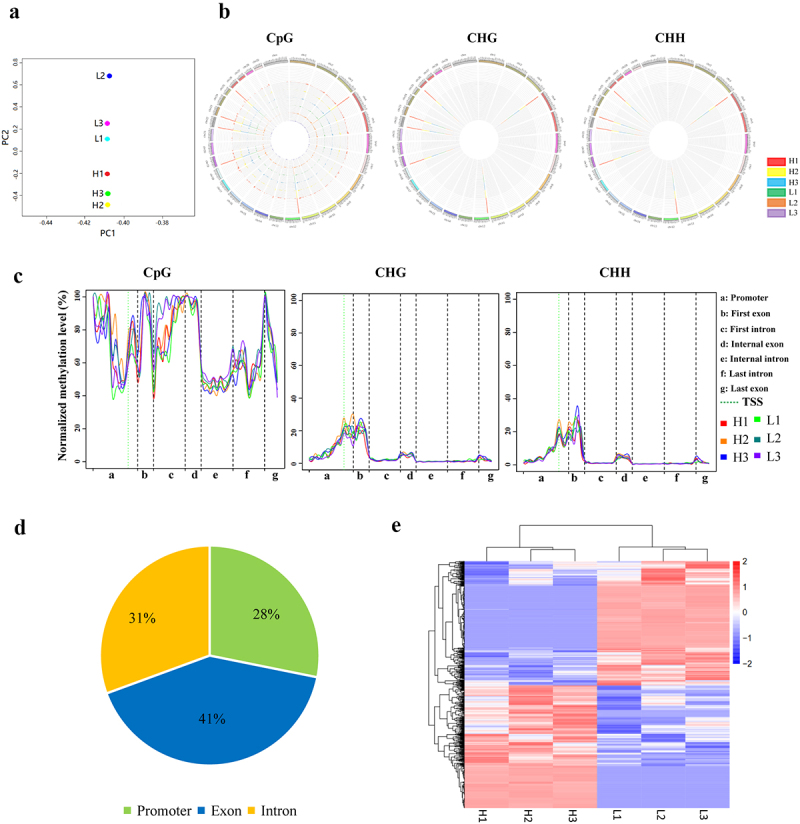
**Overview of sperm DNA methylation and differential DNA methylation regions between the H and L groups**. (a) PCA analysis on RRBS data of the H and L groups. (b) Distribution of 5mC in chromosomes. The colour from inside to outside the circle represents the sample order: H1, H2, H3, L1, L2, and L3. (c) Methylation ratio of mCpG, mCHG, and mCHH in gene elements. (d) Statistical analysis of gDMRs in the promoter, exon, and intron of genes. The percentage indicates the proportion of each kind of DMRs in the total number of DMRs. (e) Heatmap of gDMRs. Pink and blue bars represent the upregulated and downregulated methylation levels, respectively.

Three pairs of full-sibling Holstein bulls with high and low sperm motility were selected to analyse the relationship between DNA methylation and sperm motility. RRBS produced approximately 303 million clean reads and 24.2 Gb of high-quality data (Table S3). The high-quality clean reads were mapped onto the cattle reference genome (Bos taurus UMD3.1, Table S4). PCA analysis showed that PC2 could clearly distinguish H from the L group, although differences were observed among individuals (Figure 1a). The distribution of 5mC in chromosomes of bulls is shown in Figure 1b. CpG methylation modification was dominant in DNA methylation types, but CHG and CHH methylation also existed on the bull sperm genome (Figures 1B–C).

A total of 2308 DMRs that differentially methylated regions in the sperm DNA between the H and L groups were obtained (*P* < 0.05, |log2(FC)|≥1), including 948 DMRs located in 873 genes (referred to as gDMRs), 1354 DMRs located in repeat elements, and six DMRs located in microRNA regions. About 28% of gDMRs were found in the gene promoter, 41% in exon, and 31% in intron (Figure 1d, Table S5). The heatmap based on gDMRs showed that the DNA methylation patterns of three individuals within the same group were similar, showing good repeatability, whereas the overall gene methylation pattern of the H group was significantly different from that of the L group (Figure 1e).

Gene Ontology enrichment analysis revealed that gDMR-related genes (DMGs) were involved in several molecular functions, including RNA transcription (GO:0006351), cell adhesion (GO:0007155), cell differentiation (GO:0030154), and calmodulin binding (GO:0005516; *P* < 0.05, Fig. S2A). Focal adhesion, MAPK, and calcium signalling pathways were enriched by KEGG pathway analysis or Pathview (https://pathview.uncc.edu/home; Fig. S2B and Fig. S3).

### Sperm-motility-related gDMRs may be associated with as events

Alternative splicing events of sperm transcriptome between the H and L group were compared. In total, 1876 AS events in 874 genes were obtained, and the resulting transcripts were significantly and differentially expressed between the H and L groups (*P* < 0.05, |log2(FC)|≥1). Alternative TSS and alternative TTS accounted for 39.5% and 32.3%, respectively, followed by exon skipping (SKIP, 14.4%) and AE ends (8.9%, Fig. S4). This result indicated the critical role of abundant AS events during bull sperm maturation and motility.

**Figure 2.**
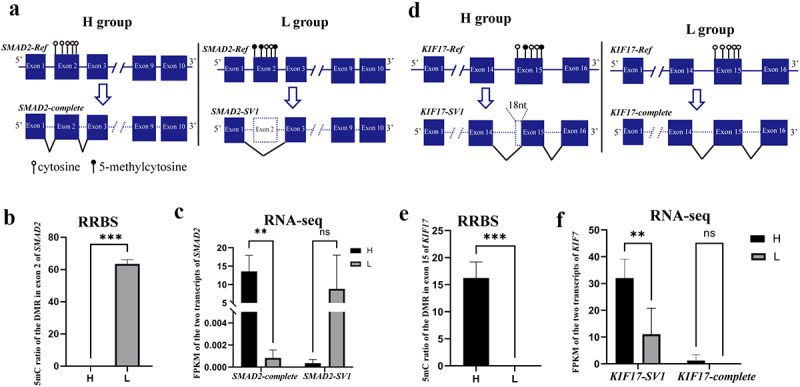
**gDMR in *SMAD2* and *KIF17* is related to AS events**. (a) Schematic diagram of the relationship between gDMR and alternative splicing of exon 2 of *SMAD2*. (b) DNA methylation level in exon 2 of *SMAD2* in the H and L groups. (c) Expression of *SMAD2-complete* and *SMAD2-SV1* of bull sperm from RNA-seq. (d) Schematic diagram of the relationship between gDMR and alternative splicing of exon 15 of *KIF17*. (e) DNA methylation level in exon 15 of *KIF17* in the H and L groups. (f) Expression of *KIF17*-complete and *KIF17-SV1* of bull sperm from RNA-seq. *P < 0.05, **P < 0.01, ***P < 0.001. ‘ns’ represents *P* > 0.05.

Interestingly, AS events were observed in gDMRs, and 781 (89%) gDMRs exhibited AS events. Of which, 123 AS transcripts within 57 genes were significantly and differentially expressed between the H and L groups (*P* < 0.05, |log2(FC)|≥1), including spermatogenesis-related genes, such as *SMAD2, KIF17, RAB22A, CCDN1*, and *PBRM1* (Figure 2, Table S6). *SMAD2* can be activated by Nodal signalling, and it can promote the proliferation of mouse spermatogonial stem/progenitor cells [37]. One DMR in exon 2 of *SMAD2* had a lower methylation level in H than in the L group (Figures 2A and 2B, Table S6). Furthermore, *SMAD2-complete* was more highly expressed in H than in the L group (*P* < 0.05), whereas *SMAD2-SV1* (exon 2 deletion) was more highly expressed in L than in the H group, but this difference was not significant (Figures 2A and 2C). These results indicated that the DNA methylation level of exon 2 of *SMAD2* may affect its AS (Figures 2A–C). Similarly, *KIF17* was specifically expressed in mouse testis and localized primarily in the principal piece of the sperm tail [38]. The 5mC ratio in exon 15 of *KIF17* was higher in H than in the L group (Figures 2D and 2E). Furthermore, *KIF17-complete* was more highly expressed in H than that in the L group (*P* < 0.05), but the expression of *KIF17-SV1* (18 bp deletion of exon 15) had no difference between the H and L groups (figure 2f). These results indicated that sperm DNA methylation plays an important role in spermatogenesis by modulating gene AS.

### High methylation level in exon 29 of PBRM1 may promote the generation of alternatively spliced transcripts

**Figure 3.**
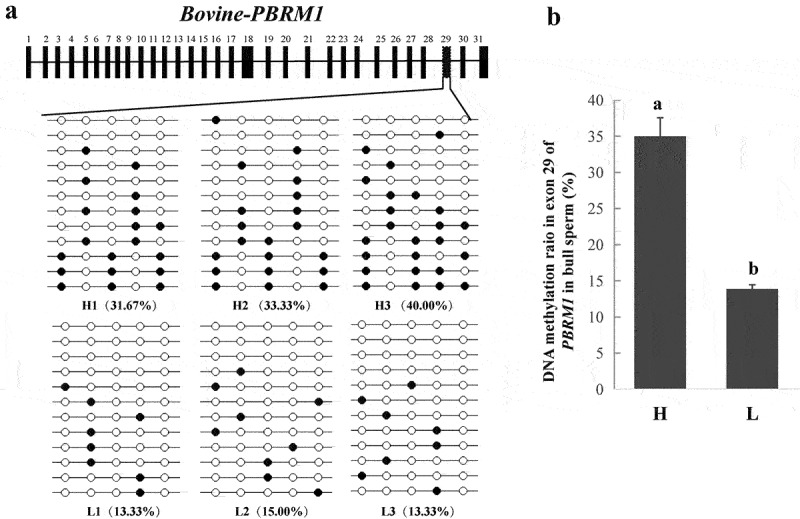
**Differential methylation in exon 29 of *PBRM1***. (a) *PBRM1* gene structure and validation of the DNA methylation level in exon 29 using BSP (six samples). (b) DNA methylation ratio of *PBRM1* between the H and L groups. Means with different lowercase letters within the same column are different (*P* < 0.05).

PBRM1, encoding a subunit of ATP-dependent chromatin-remodelling complexes, is required to stabilize the SWI/SNF chromatin remodelling complex, which is involved in mouse spermatogenesis [39–41]. Therefore, PBRM1 might play an essential role in bovine spermatogenesis. In our study, one DMR (chr22: 48,830,591–48,830,747) located in exon 29 of *PBRM1* should be further studied because the DMR was hypermethylated in the sperm of bulls from the H group, and the 5mC ratio was 74%, which was the highest among all gDMRs (Table S5). The BSP results indicated that the H group had a higher methylation level in a partial region of exon 29 than the L group (*P* < 0.05, Figures 3A and 3B), which were consistent with the results obtained from the RRBS. Furthermore, three alternative transcripts of *PBRM1* were obtained from sperm transcriptome between the H and L groups (Table S6).

**Figure 4.**
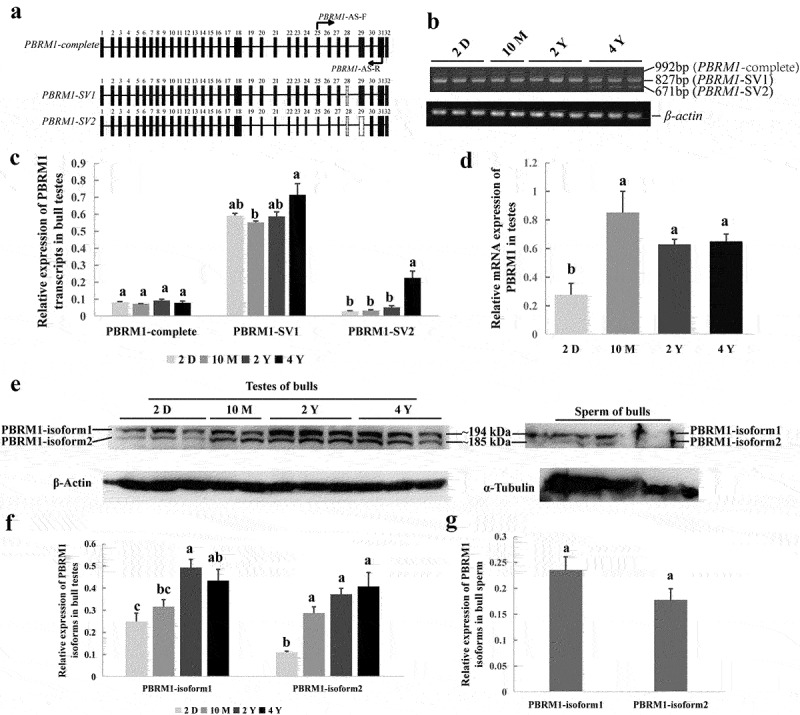
**Validation of alternative splicing in exon 29 of *PBRM1.*** (a) Schematic diagram of three spliced transcripts (*PBRM1-complete, PBRM1-SV1*, and *PBRM1-SV2*) around exon 28 and 29 of *PBRM1* (Bos_Taurus UMD3.1). (b) Three spliced transcripts of *PBRM1* in bull testis were verified by RT-PCR using *PBRM1*-AS-F/R primers. (c) qRT-PCR results of the relative mRNA expression of *PBRM1-complete, PBRM1-SV1*, and *PBRM1-SV2* in bull testes. (d) qRT-PCR results of the total mRNA expression of *PBRM1* at different stages of sexual maturity. (e) Western blot results of PBRM1 protein expression in bull testes and sperm, and β-actin and α-tubulin used as the control. (f) Expression of PBRM1 isoforms in bull testes at different stages of development. (g) Expression of PBRM1 isoforms in bull sperm. Means with different lowercase letters within the same column represent significant differences (*P* < 0.05). 2 D represents the testes of 2-day-old bulls; 10 M represents the testes of 10-month-old bulls; 2 Y represents the testes of 2-year-old bulls; 4 Y represents the testes of 4-year-old bulls.

The bovine *PBRM1* gene includes 32 exons and 31 introns. Here, exon skipping events near exon 29 of the bovine *PBRM1* gene was observed in the NCBI database (Figure 4a). Therefore, we hypothesized that DNA methylation of exon 29 would affect AS of *PBRM1*. Consequently, three transcripts, namely, *PBRM1-complete, PBRM1-SV1* (exon 28 deletion), and *PBRM1-SV2* (exons 28–29 deletion), were identified in bull testes at all stages of sexual maturity (Figure 4b and Supplemental material_original images). *PBRM1-SV1* was the primary transcript in different development stages of testes, and the expression level of *PBRM1-SV2* gradually increased with age (Figure 4c). *PBRM1-complete* was rarely and stably expressed across all stages of bull testes (Figure 4c). Compared with newborn bulls, the mRNA expression level of *PBRM1* in the testes of sexually mature bulls was significantly increased (Figure 4d). These results indicate that the expression of *PBRM1*-spliced transcripts may be related to testicular development and spermatogenesis in bulls.

Two spliced isoforms of PBRM1, namely, PBRM1-isoform1 and PBRM1-isoform2, were detected in bull testes and sperm by Western blot (Figure 4e and Supplemental material_original images). PBRM1-isoform1 and PBRM1-isoform2 corresponding to *PBRM1-SV1* and *PBRM1-SV2* were predicted with a molecular weight of approximately 194 and 185 kD, respectively, via SMS2 (https://www.detaibio.com/sms2/protein_mw.html). Furthermore, the expression level of PBRM1-isoform1 was higher than PBRM1-isoform2 in the testes and sperm; however, the expression level of PBRM1-isoform2 in the testes significantly increased with age (figure 4f). No significant difference was observed between PBRM1-isoform1 and PBRM1-isoform2 in sperm (Figure 4g). This finding indicates that the generation of *PBRM1-SV2* plays an important role in spermatogenesis. Therefore, we speculate that DNA methylation of exon 29 of bovine *PBRM1* may affect the AS of exon 29 during spermatogenesis.

### PBRM1 is located in the redundant nuclear envelope (RNE) of bull sperm, and the absence of expression affects sperm motility caused by sperm tail breakage

**Figure 5.**
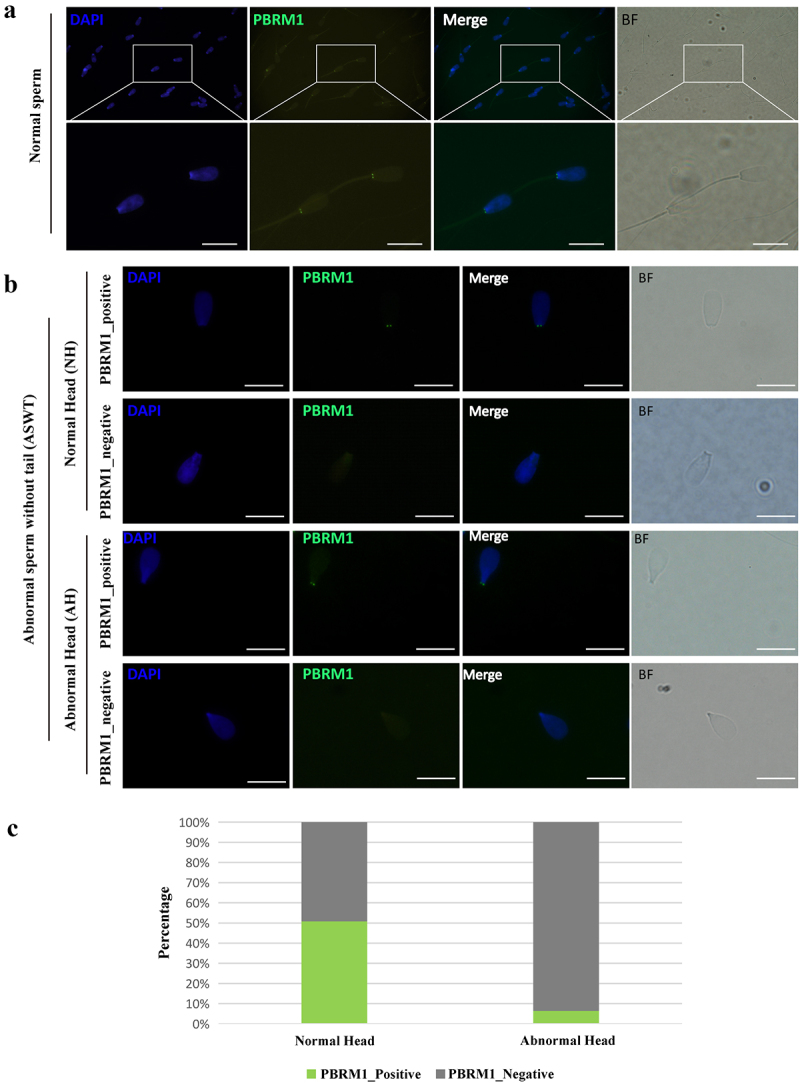
**Expression and localization of PBRM1 in sperm of adult bull**. (a) PBRM1 is located in the head-to-tail connection of normal sperm of bull. The sperm head was stained with DAPI (blue). PBRM1 was detected by anti-PBRM1 antibody (Green). BF, bright field. Scale bar: 20 µm. (b) The immunofluorescence test showed four types of sperm, including ASWT_NH_PBRM1_positive, ASWT_NH_PBRM1_negative, ASWT_AH_PBRM1_positive, and ASWT_AH_PBRM1_negative. ASWT: abnormal sperm without tail. NH: normal heads. AH: abnormal head. Scale bar: 20 µm. (c) Comparison of the percentage of the four types of sperm. The percentage of AH_PBRM1_Negative sperm was higher than AH_PBRM1_Positive. Chi-square test, *P* < 0.05.

Immunofluorescence staining with anti-PBRM1 antibodies revealed two prominent signals in the head-to-tail connection of all normal bull sperm, which were symmetrical on both sides (Figure 5a). Abnormal sperm without a tail (ASWT) was verified as normal heads (NH) or abnormal head (AH) by DAPI (4´,6-diamidino-2-phenylindole) staining and bright-field photography. These abnormal sperm were divided into four categories based on whether the sperm head morphology was normal and whether PBRM1 was expressed: ASWT_NH_PBRM1_positive, ASWT_NH_PBRM1_negative, ASWT_AH_PBRM1_positive, and ASWT_AH_PBRM1_negative (Figure 5b). The statistical results showed that ASWT_NH_PBRM1_negative sperms (n = 343) accounted for 49% of the total ASWT_NH sperms (n = 679), whereas ASWT_AH_PBRM1_negative sperms (n = 338) accounted for 94% of the total ASWT_AH sperms (n = 361, Figure 5c). Most abnormal spermatozoa had no PBRM1 expression, and sperm tails were broken. The loss of PBRM1 in the head-to-tail connection of sperm is significantly associated with sperm tail breakage (*P* < 0.05), thereby affecting sperm motility.

**Figure 6.**
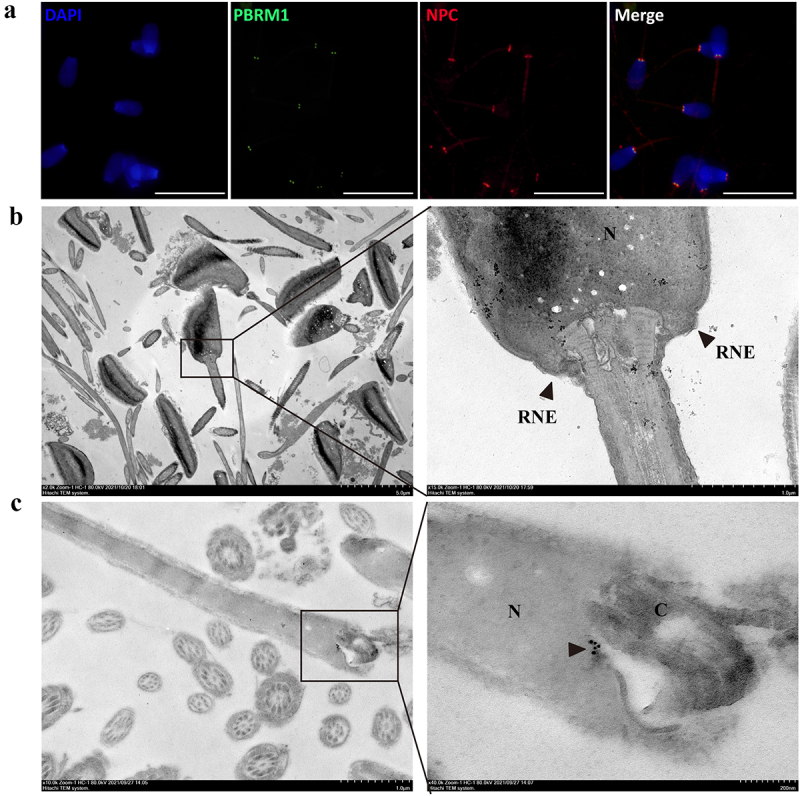
**PBRM1 proteins are enriched in the region of the redundant nuclear envelope in bovine sperm**. (a) PBRM1 (green) is significantly colocalized with NPC (red) in bull sperm. The scale bar is 20 μm. (b) Ultrastructure of the head-to-tail connection region of bull sperm by transmission electron microscopy. The RNE structure of bull sperm was highlighted by the black arrow. (c) Immunogold labelling of bull sperm with an anti-PBRM1 antibody. Positive staining in the head-to-tail connection area was highlighted by the black arrow. N, nuclei; C, centriole.

Immunofluorescence staining showed that PBRM1 is significantly colocalized with the NPC protein, which serves as a marker of the RNE in bull sperm (Figure 6a). Transmission electron microscopy showed two symmetrical redundant nuclear membranes in the head-to-tail connection of sperm (Figure 6b). Furthermore, immunoelectron microscopy showed that the colloidal gold signals to anti-PBRM1 antibody were relatively concentrated and located in the head-to-tail connection of bull sperm (Figure 6c). The results indicate that PBRM1 is expressed in the RNE of bull sperm.

## Discussion

DNA methylation and RNA transcriptome analyses were used to screen the differential DNA methylation regions of full-sibling Holstein bulls with high and low sperm motilities and to identify the pathways and genes related to sperm motility. Here, 2308 DMRs (including 948 gDMRs, 1354 repDMRs, and six miDMRs), which are involved in RNA transcription, cell adhesion, cell differentiation, and calcium ion binding, are identified. Furthermore, we highlighted that the alteration in DNA methylation and AS of *PBRM1* were associated with the structure and motility of bovine sperm. These results provide valuable data for future biomedical research and genomic and epigenomic studies of semen quality, which can be used to uncover the molecular basis underlying the economic value of sperm quality traits in bulls.

The sperm DNA methylation profile is particularly important because it is one of the factors that control the expression of imprinted genes, which are essential for foetal development and foetal growth [42]. One of the biggest concerns is that abnormal sperm epigenetic defects may be passed onto offspring and may affect their susceptibility to disease [43]. Considerable evidence has pointed out the effects of DNA methylation on male fertility and semen quality. For example, abnormalities of DNA methylation are associated with human sperm parameters, including sperm concentration, motility, and morphology [44]. Global sperm DNA methylation is associated with sperm concentration and sperm motility, but it is not associated with sperm vitality or morphology [45]. Sperm cells of low-fertility bulls have a less dense chromatin structure, higher levels of DNA damage, and higher methylation levels than those of high-fertility bulls [46]. In our study, a strong association was observed between global sperm DNA methylation and sperm motility in full-sib Holstein bulls.

Twin models are more advantageous in epigenetic studies because they share almost all genetic variations and many environmental factors, and they form natural matching controls [47]. Several studies have reported the relationship between genome-wide DNA methylation profiles and sperm quality and identified several DMRs implicated in motility by using the twins as a study model. For example, by using methyl-sensitive enrichment and microarray analyses, 580 differentially methylated loci, including fertility-related QTLs, have been identified in four pairs of monozygotic twin bulls, which have daughters with incongruous diverging performances [18]. The analyses of interindividual variations of 28 bull sperm DNA methylation and whole-genome BSP data indicate that the variably methylated regions are related to sperm motility and reproduction [23]. A total of 528 DMRs associated with embryonic development, organ development, reproduction, and the nervous system were obtained in two monozygotic twin bulls with moderately different sperm qualities [48]. In the present study, 948 gDMRs encompassing 837 genes were enriched in the RNA transcription, cell adhesion, cell differentiation, and calmodulin-binding GO terms. KEGG pathway analysis has identified pathways involved in spermatogenesis and sperm motility. For example, the focal adhesion pathway (24 DMGs) contributes to the acrosome integrity and remodelling of the cytoskeleton during sperm capacitation and spermatid adhesion [49,50]. The MAPK signalling pathway (26 DMGs) regulates the dynamics of tight and adhesion junctions and the proliferation and meiosis of germ cells [51]. The calcium signalling pathway (21 DMGs) regulates the sperm motility of mammals by controlling the calcium ion concentration and activity of calcium-dependent proteins [52]. These findings indicate that the divergence of the sperm motility of full-sibling bulls may be due to differential methylation of multiple genes participating in the critical signalling pathways related to spermatogenesis and sperm maturation.

Spermatogenesis is a highly coordinated process that requires tightly regulated gene expression programmed by epigenetic modifiers, including DNA methylation and chromatin remodelling [53]. DNA methylation plays a crucial role in correct sperm functionality. Methylation variation in genes is functionally related to sperm DNA organization and maintenance [15]. In this study, we found that the significant DMR harbours exon 29 of *PBRM1*, which is related to sperm motility. Furthermore, *PBRM1* encodes a subunit of ATP-dependent chromatin-remodelling complexes, which are required to stabilize the SWI/SNF chromatin remodelling complex [40,54,55]. The SWI/SNF chromatin remodelling complex is involved in the spermatogenesis and deficiency of the catalytic subunit of the SWI/SNF chromatin remodelling complex, which results in a meiotic arrest during mouse spermatogenesis [39,41].

Furthermore, we found that the mRNA expression level of *PBRM1* is higher in adult bull testis than in newborn bulls. The expression level of *PBRM1-SV1* and *PBRM1-SV2* was also significantly higher than that of *PBRM1*-complete in the testis. DNA methylation in promoter regions is a well-characterized epigenetic marker negatively related to gene expression regulation. However, *PBRM1* expression is positive with DNA methylation in exon. Interestingly, emerging evidence has shown that DNA methylation also regulates gene AS. Intragenic DNA methylation operates in exon to modulate alternative RNA splicing [24,56]. A recent study has demonstrated that changes in the methylation pattern of alternatively spliced exons, but not constitutively spliced exons or introns, altered inclusion levels using deactivated endonuclease Cas9 fused with enzymes that methylate or demethylate [57]. In the present study, two splice variants, namely, *PBRM1-SV1* and *PBRM1-SV2*, are considered primary transcripts, and they are highly expressed in adult bull testes. This finding indicates that DNA methylation of exon 29 in *PBRM1* results in AS of *PBRM1* and generates the high expression of splice variants, which warrants further investigation. In addition, we first found that PBRM1 was located in the RNE of bull sperm. The RNE contains an intracellular Ca^2+^ store, which can provide Ca^2+^ for the axoneme to enhance sperm motility during hyperactivation [58]. We observed that the lack of PBRM1 in sperm is associated with sperm tail breakage, indicating that PBRM1 is related to sperm motility. We successfully identify an example that DNA methylation alteration at specific loci regulates gene splicing and expression, thereby altering sperm structure and motility.

It should be pointed out that one limitation of this study is that we used only motility and did not measure other phenotypic parameters, such as membrane and DNA integrity which are important factors in sperm quality. Using these parameters can be more informative and potentially reveal more interesting results.

## Conclusions

Our findings indicate that alternations of DNA methylation and RNA AS affect sperm motility in Holstein bull; thus, investigating epigenetic changes is important to understand the genetic mechanism during spermatogenesis. We also found that DNA methylation affects AS of *PBRM1*, which is necessary for spermatogenesis and maturation, thereby generating phenotypic diversity.

## Supplementary Material

Supplemental Material

## Data Availability

The raw data of RRBS has been uploaded to the NCBI SRA, and the accession number was PRJNA818321.

## References

[1] Druet T, Fritz S, Sellem E, et al. Estimation of genetic parameters and genome scan for 15 semen characteristics traits of Holstein bulls. J Anim Breed Genet. 2009;126(4):269–16. 19630877 10.1111/j.1439-0388.2008.00788.x

[2] Mathevon M, Buhr MM, Dekkers JC. Environmental, management, and genetic factors affecting semen production in Holstein bulls. J Dairy Sci. 1998;81(12):3321–3330. 9891279 10.3168/jds.S0022-0302(98)75898-9

[3] Lessard C, Masseau I, Bilodeau JF, et al. Semen characteristics of genetically identical quadruplet bulls. Theriogenology. 2003;59(8):1865–1877. 12566158 10.1016/s0093-691x(02)01256-6

[4] Snoj T, Kobal S, Majdic G. Effects of season, age, and breed on semen characteristics in different *bos Taurus* breeds in a 31-year retrospective study. Theriogenology. 2013;79(5):847–852. 23380262 10.1016/j.theriogenology.2012.12.014

[5] Lonergan P, Fair S. Influence of bull age, ejaculate number, and season of collection on semen production and sperm motility parameters in Holstein Friesian bulls in a commercial artificial insemination centre. J Anim Sci. 2018;96(6):2408–2418. 29767722 10.1093/jas/sky130 PMC6095274

[6] Guo F, Yang B, Ju ZH, et al. Alternative splicing, promoter methylation, and functional SNPs of sperm flagella 2 gene in testis and mature spermatozoa of Holstein bulls. Reproduction. 2014;147(2):241–252. 24277870 10.1530/REP-13-0343

[7] Wang X, Yang C, Guo F, et al. Integrated analysis of mRNAs and long noncoding RNAs in the semen from Holstein bulls with high and low sperm motility. Sci Rep. 2019;9(1):2092. 30765858 10.1038/s41598-018-38462-x PMC6376035

[8] Egger G, Liang G, Aparicio A, et al. Epigenetics in human disease and prospects for epigenetic therapy. Nature. 2004;429(6990):457–463. 15164071 10.1038/nature02625

[9] Suzuki MM, Bird A. DNA methylation landscapes: provocative insights from epigenomics. Nat Rev Genet. 2008;9(6):465–476. 18463664 10.1038/nrg2341

[10] Bell JT, Spector TD. A twin approach to unraveling epigenetics. Trends Genet. 2011;27(3):116–125. 21257220 10.1016/j.tig.2010.12.005 PMC3063335

[11] Tan Q, Christiansen L, von Bornemann Hjelmborg J, et al. Twin methodology in epigenetic studies. J Exp Biol. 2015;218(Pt 1):134–139. 25568460 10.1242/jeb.107151

[12] Du Y, Li M, Chen J, et al. Promoter targeted bisulfite sequencing reveals DNA methylation profiles associated with low sperm motility in asthenozoospermia. Hum Reprod. 2016;31(1):24–33. 26628640 10.1093/humrep/dev283

[13] Boissonnas CC, Abdalaoui HE, Haelewyn V, et al. Specific epigenetic alterations of IGF2-H19 locus in spermatozoa from infertile men. Eur J Hum Genet. 2010;18(1):73–80. 19584898 10.1038/ejhg.2009.117 PMC2987171

[14] Laqqan M, Tierling S, Alkhaled Y, et al. Alterations in sperm DNA methylation patterns of oligospermic males. Reprod Biol. 2017;17(4):396–400. 29108863 10.1016/j.repbio.2017.10.007

[15] Capra E, Lazzari B, Turri F, et al. Epigenetic analysis of high and low motile sperm populations reveals methylation variation in satellite regions within the pericentromeric position and in genes functionally related to sperm DNA organization and maintenance in *Bos Taurus*. BMC Genomics. 2019;20(1):940. 31810461 10.1186/s12864-019-6317-6 PMC6898967

[16] Verma A, Rajput S, De S, et al. Genome-wide profiling of sperm DNA methylation in relation to Buffalo (Bubalus bubalis) bull fertility. Theriogenology. 2014;82(5):750–9.e1. 25023295 10.1016/j.theriogenology.2014.06.012

[17] Kropp J, Carrillo JA, Namous H, et al. Male fertility status is associated with DNA methylation signatures in sperm and transcriptomic profiles of bovine preimplantation embryos. BMC Genomics. 2017;18(1):280. 28381255 10.1186/s12864-017-3673-y PMC5382486

[18] Shojaei Saadi HA, É F, Vigneault C, et al. Genome-wide analysis of sperm DNA methylation from monozygotic twin bulls. Reprod Fertil Dev. 2017;29(4):838–843. 26751019 10.1071/RD15384

[19] Perrier J, Sellem E, Prézelin A, et al. A multi-scale analysis of bull sperm methylome revealed both species peculiarities and conserved tissue-specific features. BMC Genomics. 2018;19(1):404. 29843609 10.1186/s12864-018-4764-0 PMC5975405

[20] Zhou Y, Connor EE, Bickhart DM, et al. Comparative whole genome DNA methylation profiling of cattle sperm and somatic tissues reveals striking hypomethylated patterns in sperm. Gigascience. 2018;7(5):giy039. 29635292 10.1093/gigascience/giy039 PMC5928411

[21] Ahlawat S, Sharma R, Arora R, et al. Promoter methylation and expression analysis of Bvh gene in bulls with varying semen motility parameters. Theriogenology. 2019;125:152–156. 30447494 10.1016/j.theriogenology.2018.11.001

[22] Liu Y, Zhang Y, Yin J, et al. Distinct H3K9me3 and DNA methylation modifications during mouse spermatogenesis. J Biol Chem. 2019;294(49):18714–18725. 31662436 10.1074/jbc.RA119.010496 PMC6901318

[23] Liu S, Fang L, Zhou Y, et al. Analyses of inter-individual variations of sperm DNA methylation and their potential implications in cattle. BMC Genomics. 2019;20(1):888. 31752687 10.1186/s12864-019-6228-6 PMC6873545

[24] Lev Maor G, Yearim A, Ast G. The alternative role of DNA methylation in splicing regulation. Trends Genet. 2015;31(5):274–280. 25837375 10.1016/j.tig.2015.03.002

[25] Linker SM, Urban L, Clark SJ, et al. Combined single-cell profiling of expression and DNA methylation reveals splicing regulation and heterogeneity. Genome Biol. 2019;20(1):30. 30744673 10.1186/s13059-019-1644-0 PMC6371455

[26] Yoshimi A, Lin KT, Wiseman DH, et al. Coordinated alterations in RNA splicing and epigenetic regulation drive leukaemogenesis. Nature. 2019;574(7777):273–277. 31578525 10.1038/s41586-019-1618-0 PMC6858560

[27] Lu H, Sun F, Wang G, et al. Technical code of practice of bovine frozen semen production, NY/T 1234-2018. Ministry of Agriculture and Rural Affairs of the People’s Republic of China.

[28] Herthnek D, Englund S, Willemsen P, et al. Sensitive detection of Mycobacterium avium subspparatuberculosis in bovine semen by real-time PCR. J Appl Microbiol. 2006;100(5):1095–1102. 16630010 10.1111/j.1365-2672.2006.02924.x

[29] Gu H, Bock C, Mikkelsen TS, et al. Genome-scale DNA methylation mapping of clinical samples at single-nucleotide resolution. Nat Methods. 2010;7(2):133–136. 20062050 10.1038/nmeth.1414 PMC2860480

[30] Gu H, Smith ZD, Bock C, et al. Preparation of reduced representation bisulfte sequencing libraries for genome-scale DNA methylation profling. Nat Protoc. 2011;6(4):468–481. 21412275 10.1038/nprot.2010.190

[31] Langmead B . Aligning short sequencing reads with Bowtie. Curr. Protoc. Bioinformatics. Wiley Online Library. 2010. 10.1002/0471250953.bi1107s32 PMC3010897 21154709

[32] Akalin A, Kormaksson M, Li S, et al. Methylkit: a comprehensive R package for the analysis of genome-wide DNA methylation profiles. Genome Biol. 2012;13(10):R87. 23034086 10.1186/gb-2012-13-10-r87 PMC3491415

[33] Luo W, Brouwer C. Pathview: an R/Biocondutor package for pathway-based data integration and visualization. Bioinformatics. 2013;29(14):1830–1831. 23740750 10.1093/bioinformatics/btt285 PMC3702256

[34] Luo W, Pant G, Bhavnasi YK, et al. Pathview Web: user friendly pathway visualization and data integration. Nucleic Acids Res. 2017;45(W1):W501–W508. 28482075 10.1093/nar/gkx372 PMC5570256

[35] Dupont JM, Tost J, Jammes H, et al. De novo quantitative bisulfite sequencing using the pyrosequencing technology. Anal Biochem. 2004;333(1):119–127. 15351288 10.1016/j.ab.2004.05.007

[36] Livak KJ, Schmittgen TD. Analysis of relative gene expression data using real-time quantitative PCR and the 2(-Delta Delta C(T)) Method. Methods. 2001;25(4):402–408. 11846609 10.1006/meth.2001.1262

[37] He Z, Jiang J, Kokkinaki M, et al. Nodal signaling via an autocrine pathway promotes proliferation of mouse spermatogonial stem/progenitor cells through Smad2/3 and Oct- 4activation. Stem Cells. 2009;27(10):2580–2590. 19688838 10.1002/stem.198 PMC3443855

[38] Kotaja N, Macho B, Sassone-Corsi P. Microtubule-independent and protein kinase A-mediated function of kinesin KIF17b controls the intracellular transport of activator of CREM in testis (ACT). J Biol Chem. 2005;280(36):31739–31745. 16002395 10.1074/jbc.M505971200

[39] Kim Y, Fedoriw AM, Magnuson T. An essential role for a mammalian SWI/SNF chromatin-remodeling complex during male meiosis. Development. 2012;139(6):1133–1140. 22318225 10.1242/dev.073478 PMC3283123

[40] Kadoch C, Crabtree GR. Mammalian SWI/SNF chromatin remodeling complexes and cancer: mechanistic insights gained from human genomics. Sci Adv. 2015;1(5):e1500447. 26601204 10.1126/sciadv.1500447 PMC4640607

[41] Menon DU, Shibata Y, Mu W, et al. Mammalian SWI/SNF collaborates with a polycomb-associated protein to regulate male germline transcription in the mouse. Development. 2019;146(19):dev174094. 31043422 10.1242/dev.174094 PMC6803380

[42] Reik W, Walter J. Genomic imprinting: parental influence on the genome. Nat Rev Genet. 2001;2(1):21–32. 11253064 10.1038/35047554

[43] Wei Y, Yang CR, Wei YP, et al. Paternally induced transgenerational inheritance of susceptibility to diabetes in mammals. Proc Natl Acad Sci USA. 2014;111(5):1873–1878. 24449870 10.1073/pnas.1321195111 PMC3918818

[44] Houshdaran S, Cortessis VK, Siegmund K, et al. Widespread epigenetic abnormalities suggest a broad DNA methylation erasure defect in abnormal human sperm. PLoS One. 2007;2(12):e1289. 18074014 10.1371/journal.pone.0001289 PMC2100168

[45] Montjean D, Zini A, Ravel C, et al. Sperm global DNA methylation level: association with semen parameters and genome integrity. Andrology. 2015;3(2):235–240. 25755112 10.1111/andr.12001

[46] Narud B, Khezri A, Zeremichael TT, et al. Sperm chromatin integrity and DNA methylation in Norwegian Red bulls of contrasting fertility. Mol Reprod Dev. 2021;88(3):187–200. 33634579 10.1002/mrd.23461

[47] Bell JT, Spector TD. DNA methylation studies using twins: what are they telling us? Genome Biol. 2012;13(10):172. 23078798 10.1186/gb-2012-13-10-172 PMC3491399

[48] Liu S, Chen S, Cai W, et al. Divergence analyses of sperm DNA methylomes between monozygotic twin AI bulls. Epigenomes. 2019;3(4):21. 34968253 10.3390/epigenomes3040021 PMC8594723

[49] González-Fernández L, Macías-García B, Loux SC, et al. Focal adhesion kinases and calcium/calmodulin-dependent protein kinases regulate protein tyrosine phosphorylation in stallion sperm. Biol Reprod. 2013;88(6):138. 23595906 10.1095/biolreprod.112.107078

[50] Wong EW, Lee WM, Cheng CY. Secreted Frizzled-related protein 1 (sFRP1) regulates spermatid adhesion in the testis via dephosphorylation of focal adhesion kinase and the nectin-3 adhesion protein complex. FASEB J. 2013;27(2):464–477. 23073828 10.1096/fj.12-212514 PMC3545539

[51] Ni F, Hao S, Yang W. Multiple signaling pathways in Sertoli cells: recent findings in spermatogenesis. Cell Death Dis. 2019;10(8):541. 31316051 10.1038/s41419-019-1782-z PMC6637205

[52] Chung J, Shim S, Everley R, et al. Structurally distinct Ca(2+) signaling domains of sperm flagella orchestrate tyrosine phosphorylation and motility. Cell. 2014;157(4):808–822. 24813608 10.1016/j.cell.2014.02.056 PMC4032590

[53] Wang J, Tang C, Wang Q, et al. NRF1 coordinates with DNA methylation to regulate spermatogenesis. FASEB J. 2017;31(11):4959–4970. 28754714 10.1096/fj.201700093R PMC5636708

[54] Varela I, Tarpey P, Raine K, et al. Exome sequencing identifies frequent mutation of the SWI/SNF complex gene PBRM1 in renal carcinoma. Nature. 2011;469(7331):539–542. 21248752 10.1038/nature09639 PMC3030920

[55] Euskirchen G, Auerbach RK, Snyder M. SWI/SNF chromatin-remodeling factors: multiscale analyses and diverse functions. J Biol Chem. 2012;287(37):30897–30905. 22952240 10.1074/jbc.R111.309302 PMC3438922

[56] Maunakea AK, Chepelev I, Cui K, et al. Intragenic DNA methylation modulates alternative splicing by recruiting MeCP2 to promote exon recognition. Cell Res. 2013;23(11):1256–1269. 23938295 10.1038/cr.2013.110 PMC3817542

[57] Shayevitch R, Askayo D, Keydar I, et al. The importance of DNA methylation of exons on alternative splicing. RNA. 2018;24(10):1351–1362. 30002084 10.1261/rna.064865.117 PMC6140467

[58] Ho HC, Suarez SS. Characterization of the intracellular calcium store at the base of the sperm flagellum that regulates hyperactivated motility. Biol Reprod. 2003;68(5):1590–1596. 12606347 10.1095/biolreprod.102.011320

