# Supplementary material for: Coordinated alternation of DNA methylation and alternative splicing of PBRM1 affect bovine sperm structure and motility
Source: Epigenetics. 2023 Mar 3;18(1):2183339. doi: 10.1080/15592294.2023.2183339 (PMC9988346; doi:10.1080/15592294.2023.2183339)
Supplement: Supplemental Material [file KEPI_A_2183339_SM3491.zip › Supplementary files/Supplementary Figure Legends.docx]

## Supplementary Figures Legends

**Fig. S1.** Patterns of alternative splicing events detected by ASprofile by pairwise transcript comparisons. (A) Including or excluding of single exon (SKIP_ON/OFF) and multiple exons (MSKIP_ON/OFF). (B) Including or excluding of single (IR_ON/OFF) and multiple (MIR_ON/OFF) introns. (C) Alternative exon ends (AE). (C) Alternative transcription start site (TSS). (D) Alternative transcription termination site (TTS).

**Fig. S2.** GO and KEGG analyses of gDMRs. (A) GO enrichment analysis of DMRs related genes. (B) KEGG enrichment analysis of DMRs related genes using DAVID.

**Fig. S3.** Focal adhesion (A) Calcium (B) , and MAPK (C) signaling pathways were enriched by differentially methylated genes and were showed by Pathview. Green represents down-regulated genes of methylation, while red indicates up-regulated ones.

**Fig. S4.** Statistic of AS transcripts which were significantly and differentially expressed between the H and L group.
