# Supplementary figures and images for: Coordinated alternation of DNA methylation and alternative splicing of PBRM1 affect bovine sperm structure and motility
Source: Epigenetics. 2023 Mar 3;18(1):2183339. doi: 10.1080/15592294.2023.2183339 (PMC9988346; doi:10.1080/15592294.2023.2183339)

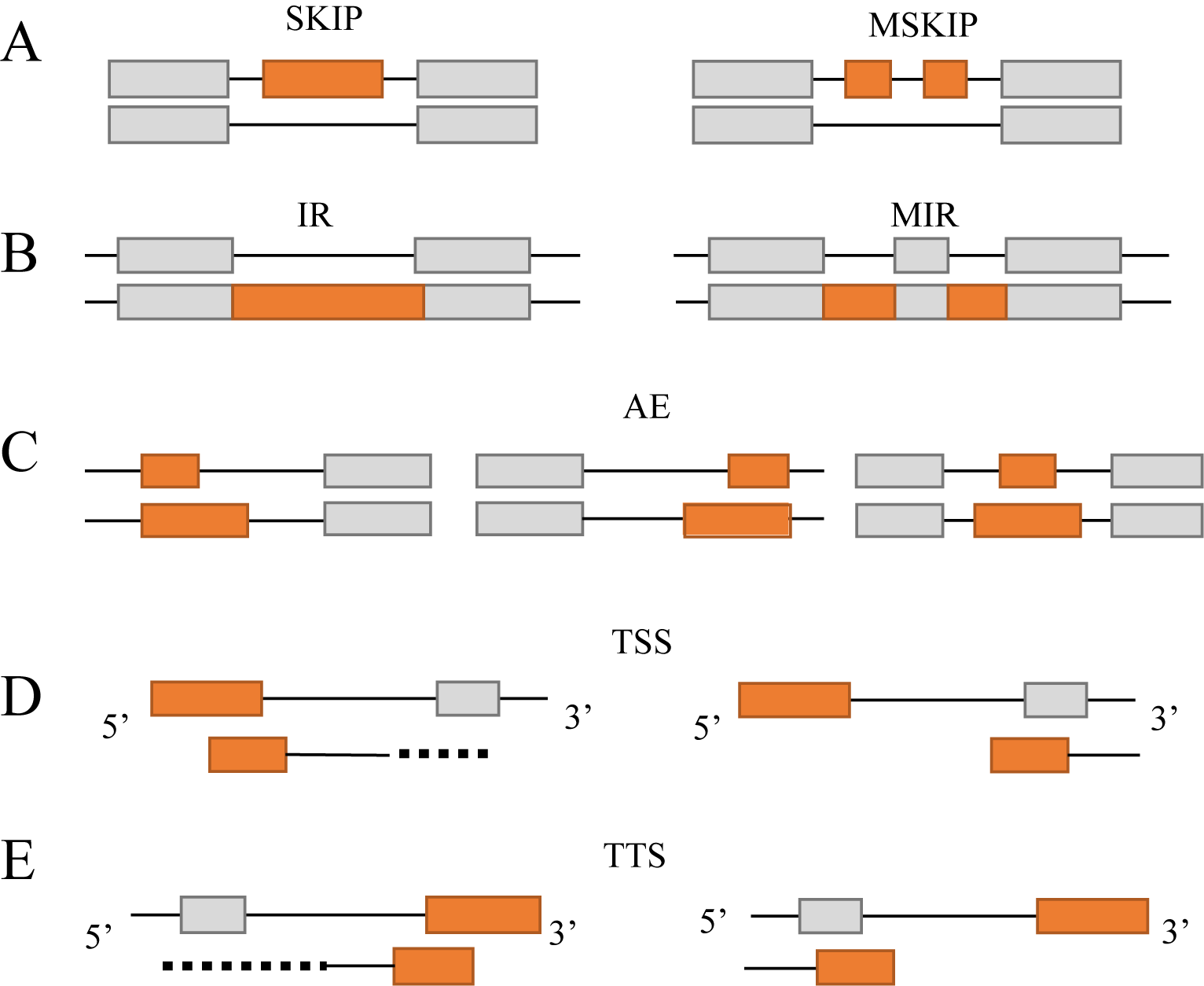

Supplement: Supplemental Material [file KEPI_A_2183339_SM3491.zip › Supplementary files/Figure S1.tif]

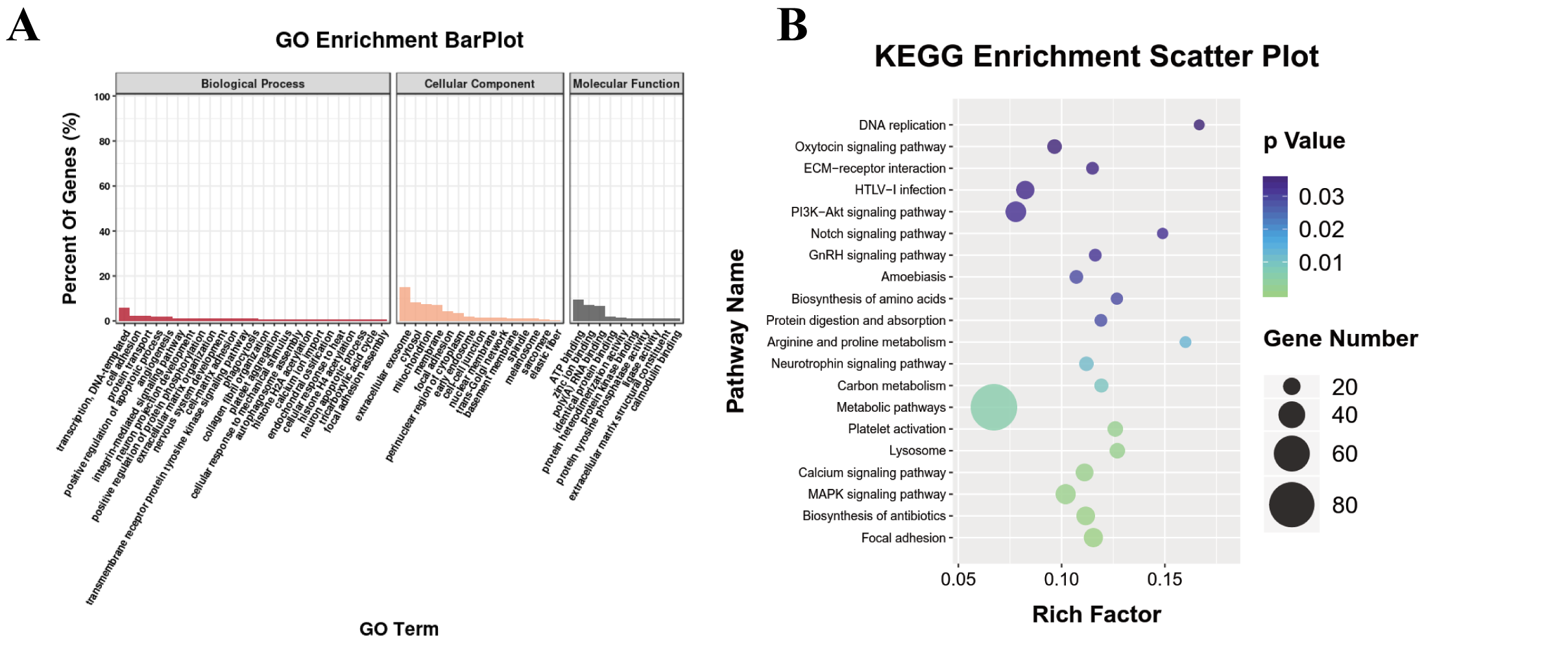

Supplement: Supplemental Material [file KEPI_A_2183339_SM3491.zip › Supplementary files/Figure S2.tif]

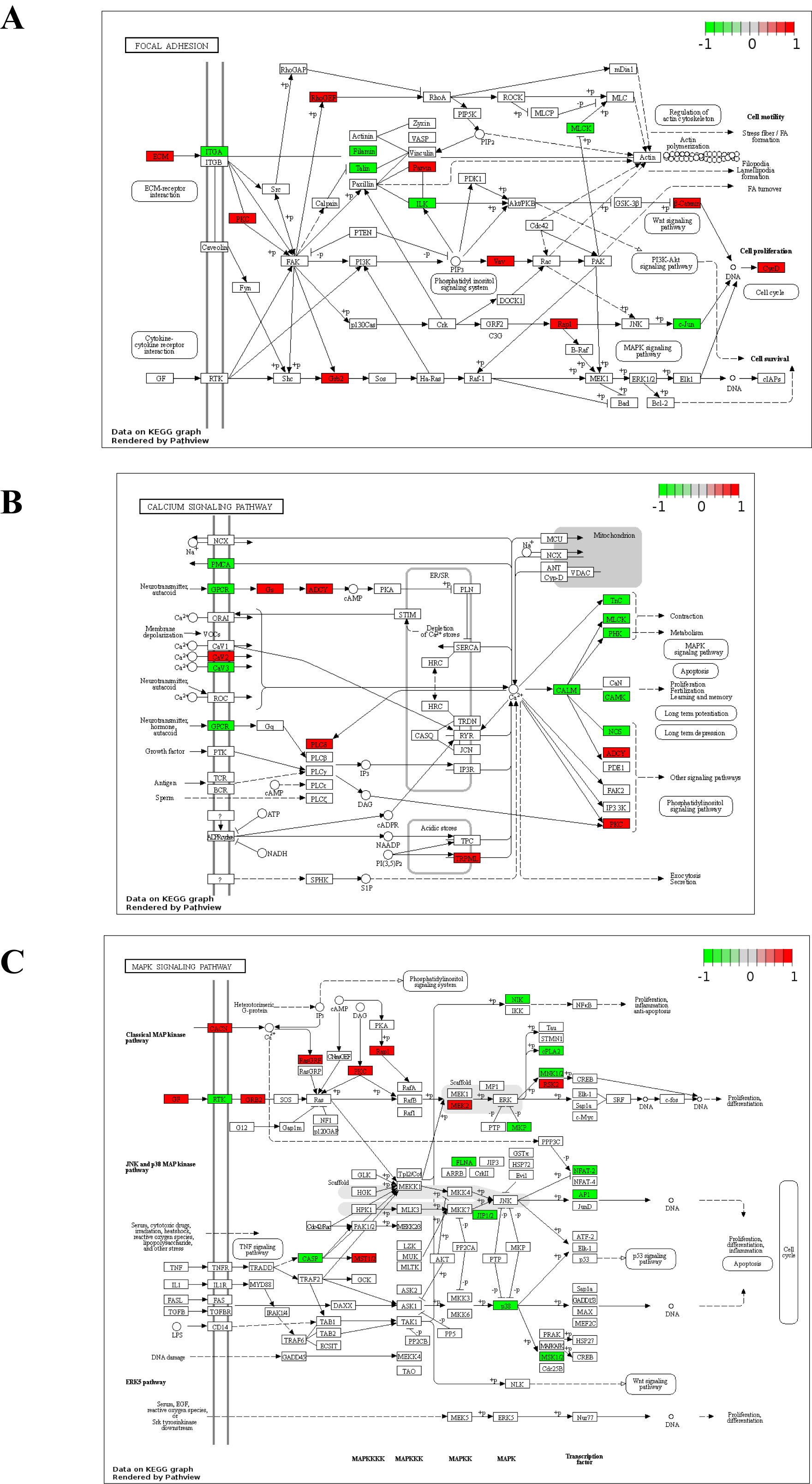

Supplement: Supplemental Material [file KEPI_A_2183339_SM3491.zip › Supplementary files/Figure S3.tif]

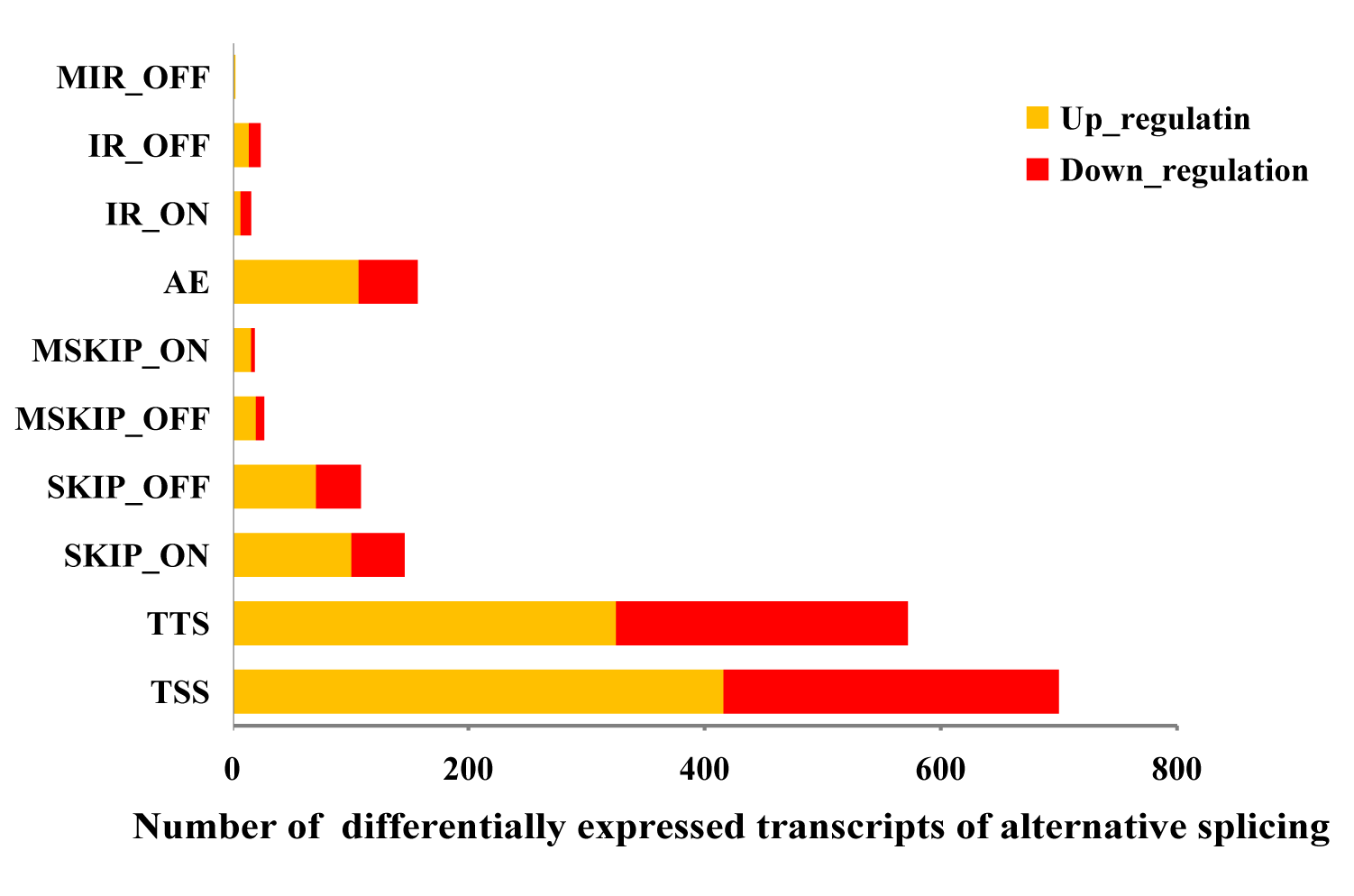

Supplement: Supplemental Material [file KEPI_A_2183339_SM3491.zip › Supplementary files/Figure S4.tif]
